# SHORT-TERM COUPLED ASSOCIATION BETWEEN BLOOD PRESSURE AND COGNITIVE FUNCTIONING

**DOI:** 10.1093/geroni/igac059.2061

**Published:** 2022-12-20

**Authors:** Tomiko Yoneda, Andrea Piccinin, Jonathan Rush, Nathan Lewis, Jamie Knight, Rebecca Vendittelli, Scott Hofer

**Affiliations:** Northwestern University, Evanston, Illinois, United States; University of Victoria, Victoria, British Columbia, Canada; University of Victoria, Victoria, British Columbia, Canada; University of Victoria, Victoria, British Columbia, Canada; University of Victoria, Victoria, British Columbia, Canada; University of Victoria, Victoria, British Columbia, Canada; University of Victoria, Victoria, British Columbia, Canada

## Abstract

An extensive body of research has investigated the association between cognition and blood pressure (BP). Limited research, however, has examined the association at the daily within-person level. No study has yet applied an intensive measurement design utilizing the convenience and objectivity of mobile devices for cognitive assessments and automated BP monitors. To address this gap in the literature, we recruited community-residing healthy adults (N=64; Mage=70.6, SD=3.5; 76.6% female), who recorded their BP and completed a battery of brief cognitive tasks twice daily for 14 days. Multi-level models estimated associations between systolic/diastolic BP (sBP/dBP) and cognitive functioning, adjusting for time-varying self-reported effort, stressors, and physical activity, as well as time-invariant variables (age, sex, education, and anti-hypertensive medication). At the interindividual level, results suggest that individuals with higher overall sBP and dBP relative to others performed worse on delayed-reproduction and visual short-term memory tasks. At the intraindividual level, on occasions when individuals had higher sBP compared to their personal average, they performed relatively worse on delayed-reproduction tasks. Mean sBP was also significant within these models, suggesting that this coupled relationship is stronger for individuals with higher sBP on average. Analyses did not indicate significant associations for working memory, reaction time, or cognitive interference assessments, which may have been due to relatively limited power for between-person analyses. Use of such digital health technology is critical for detecting the complex nature of and interplay between physiological and cognitive processes. Further, research based on intraindividual associations may contribute to strategies aiming to promote lifestyle modifications.

